# A Review on Microbial Electrocatalysis Systems Coupled with Membrane Bioreactor to Improve Wastewater Treatment

**DOI:** 10.3390/microorganisms7100372

**Published:** 2019-09-20

**Authors:** Jicun Wang, Shuai Zhao, Apurva Kakade, Saurabh Kulshreshtha, Pu Liu, Xiangkai Li

**Affiliations:** 1 Gansu Key Laboratory of Biomonitoring and Bioremediation for Environment Pollution, School of Life Science, Lanzhou University, 222 South Tianshui Rd, Lanzhou 730000, China; wangjc17@lzu.edu.cn (J.W.); zhaosh15@lzu.edu.cn (S.Z.); 2 Faculty of Applied Sciences and Biotechnology, Shoolini University of Biotechnology and Management Sciences, Bajhol, Solan, Himachal Pradesh 173229, India; apurva2017@lzu.edu.cn (A.K.); sourabhkulshreshtha@shooliniuniversity.com (S.K.); 3 Department of Developmental Biology, School of Life Sciences, Lanzhou University, Tianshuinanlu #222, Lanzhou 730000, China; liupu@lzu.edu.cn

**Keywords:** microbial electrocatalysis system, membrane bioreactor, microbial electrolytic cell, microbial fuel cell, membrane fouling, wastewater treatment

## Abstract

Microbial electrocatalysis is an electro reaction that uses microorganisms as a biocatalyst, mainly including microbial electrolytic cells (MEC) and microbial fuel cells (MFC), which has been used for wastewater treatment. However, the low processing efficiency is the main drawback for its practical application and the additional energy input of MEC system results in high costs. Recently, MFC/MEC coupled with other treatment processes, especially membrane bioreactors (MBR), has been used for high efficiency and low-cost wastewater treatment. In these systems, the wastewater treatment efficiency can be improved after two units are operated and the membrane fouling of MBR can also be alleviated by the electric energy that was generated in the MFC. In addition, the power output of MFC can also reduce the energy consumption of microbial electrocatalysis systems. This review summarizes the recent studies about microbial electrocatalysis systems coupled with MBR, describing the combination types and microorganism distribution, the advantages and limitations of the systems, and also addresses several suggestions for the future development and practical applications.

## 1. Introduction

In recent years, with the development of industry and human living standards, new types of wastewater appear constantly and the traditional wastewater treatment technologies cannot meet increasingly stringent discharge standards [1]. There are several new wastewater treatment technologies and microbial electrocatalysis systems have been especially developed for wastewater treatment. A microbial electrocatalysis system is a promising technology that uses microorganisms as biocatalysis to convert chemical energy into other forms, such as electricity, hydrogen, and methane [2]. In general, microbial electrocatalysis mainly includes microbial electrolytic cells (MEC) and microbial fuel cells (MFC) [3,4]. The similarity between MEC and MFC is the use of electrodes and microorganisms as core elements and energy can be recovered from wastewater by the catalytic action of microorganisms, which the pollutant removal and energy recovery can receive simultaneously without secondary pollution [5,6]. Therefore, microbial electrocatalysis systems are expected to become new future technologies in the field of wastewater treatment.

MFC is a spontaneous reactor that mainly uses microorganisms to degrade organic matter and generate electricity at the same time, without requiring energy to drive the reaction. It is even effective as a biosensor for real-time monitoring of pollutants in wastewater [7]. In addition, MFC has less sludge generation than other conventional wastewater treatment methods [8]. The microorganisms play an important role in MFC performance. Several researchers have investigated that *Proteobacteria, Firmicutes*, and *Bacteroidetes* are the main phyla in MFC [9]. *Proteobacteria* always exists in MFC with different carbon sources, which can degrade organic matter and mediate electron transfer. *Firmicutes* and *Bacteroidetes* also play an important role in the degradation of organic matter and electron transfer [10]. Although it has many significant advantages, the practical application of MFC is limited mainly because MFCs showcase low processing efficiency, making it difficult to meet the emission standards [11]. Several studies reported that single-chamber MFC was used to treat olive mill wastewater, in which the total chemical oxygen demand (COD) and biochemical oxygen demand (BOD_5_) decreased by 65% and 50%, respectively [10]. In addition, MFC has also been used in the domestic wastewater treatment, as follows: When hydraulic retention time (HRT) was 1.1 h, there was a 42% removal of COD; when HRT was 4 h, 79% of COD was removed. However, a large amount of COD remained in the water, which cannot be discharged directly though the MFC treatment. Therefore, using a MFC system alone would not recycle wastewater [12].

MEC is a non-spontaneous reactor that should apply voltage to the cell to drive a bioelectrochemical reaction (e.g., voltage input: 0.1–0.6 V) [2]. Recently, MEC has been studied to treat wastewater that can produce hydrogen and reduce metals [13,14]. A previous study reported that proper electrical stimulation can promote the metabolism of microorganisms and accelerate microorganism growth [15]. This might be due to the fact that an electric current can enhance the extracellular secretion and contribute to the formation of biofilms [16]. Although MEC has been shown to be able to recover energy and treat wastewater, it requires the input of electricity to start the reaction, which leads to high costs. The research showed that a pilot-scale MEC was operated for one year to treat domestic wastewater, which only achieved an average of 34% COD removal [17]. The microorganisms components were studied and the results showed *Pseudomonas, Shewanella*, and *Desulfovibrio* are effectual in MEC during wastewater treatment [18]. *Pseudomonas* has strong metabolic capacity and is commonly used for extracellular electron transfer. In addition, *Pseudomonas* and *Desulfovibrio* have metal resistance and organic matter degradation abilities [18].

Membrane bioreactor (MBR) has the characteristics of higher separation efficiency, high treatment quality, and greater convenience [19]. However, unavoidable membrane fouling and high energy consumptions limit its wide usage [20]. Membrane fouling not only leads to frequent cleaning and replacement of components, but also reduces the membrane life and increases the operation cost [21]. Some approaches have been developed to reduce the difficulties related to membrane fouling and high energy consumption [22], but these methods require physical and chemical reagents, which result in high operating costs [23]. Microbial electrocatalysis systems coupled with MBR to improve wastewater treatment have been studied [24,25]. Researchers found that the MFC-MBR combination system has two types and they can complement each other [26]. One system is an internal configuration in which the anode chamber submerges into the bioreactor. The cathode chamber is composed of an aeration tank of MBR [27]. The power generation by MFC can effectively alleviate membrane fouling and partially offset MBR energy consumption [11]. The other system has an external configuration in which the MFC-MBR system is not truly integrated and has stages that process the wastewater from one stage to another [28]. MEC applied to MBR might mitigate membrane fouling. Additionally, the voltage input to MEC-MBR system has two different effects on microorganisms. One is the electric current generation that stimulates microbial growth and degrades the pollutants and the other is that the excess electricity may inhibit microbes. This is primarily reflected in the permeability of the membrane. If membrane permeability becomes inappropriate, the material and energy channels may be disordered and it may lead to decreased microbial activity or even apoptosis [29]. Several studies reported that different voltages were applied in a system to research the effects on COD removal and more than 95% of the COD was eliminated when the COD was 320 mg/L. The results showed that about 71% of substrate energy was recovered when the applied voltage was 0.7 V [30]. Hence, a MEC-MBR system might have broad prospects in the wastewater treatment field.

This aim of this review is to discuss the performance of microbial electrocatalysis systems coupled with MBR by describing different integration types, along with summarizing the strengths and weaknesses of each integration type and an analysis of the microbial distribution. Furthermore, several challenges for upcoming developments and practical applications are addressed. On this basis, researchers can optimize microbial electrocatalysis systems coupled with MBR operation to achieve sustainable wastewater treatment.

## 2. Role of Microbial Electrocatalysis Systems Coupled with MBR System Configurations

### 2.1. MFC-MBR System Configurations

#### 2.1.1. Internal Configuration

The internal configuration is a type of MFC-MBR system where electrochemistry is applicable within MBR. In this type of system, the membrane component was installed in the cathode chamber [27]. Compared with conventional MBR (CMBR), several advantages were presented in the MFC-MBR combined system, which included proper membrane fouling mitigation, wastewater treatment efficiency improvement, and energy consumption reduction (Table 1) [31]. The COD removal of a type of MFC-MBR system that was conducted by a conductive membrane (with Fe/Mn/C/F/O elements) (97.4%) was higher than the CMBR system (90%) due to the fact that the conductive membrane was optimized by electrical energy, which was generated by MFC [32]. In addition, electrical energy (446 mW/m^3^) was generated in this system, which can reduce energy input [33].

##### Hollow-Fiber Membrane Bioreactor and Microbial Fuel Cell

**Figure 1:**
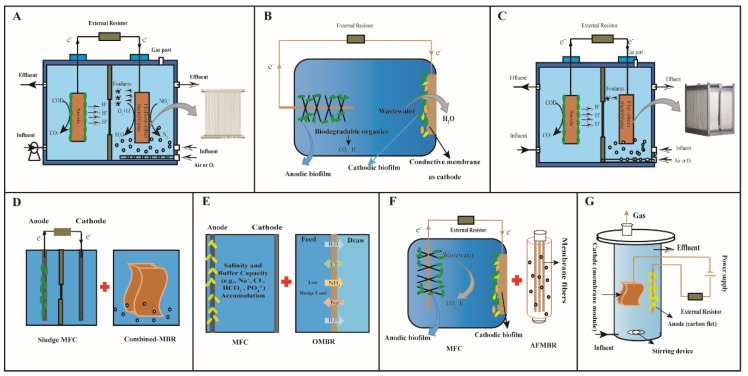
Schematic diagram of the MFC/MEC-MBR system. (**A**) Hollow-fiber membrane bioreactor and microbial fuel cell; (**B**) membrane bioreactor and air-bio cathode microbial fuel cell; (**C**) flat-sheet membrane bioreactor and microbial fuel cell; (**D**) membrane bioreactor and sludge microbial fuel cell; (**E**) osmotic membrane bioreactor and microbial fuel cell; (**F**) anaerobic fluidized bed membrane bioreactor and microbial fuel cell; (**G**) microbial electrolysis cell-anaerobic membrane bioreactor.

Hollow-fiber (HF) membrane has many size and weight advantages as compared with the other membrane separation devices. A new internal MFC-MBR system, which integrates a hollow-fiber MBR with MFC, has been developed (Figure 1A) [31]. In this combined MFC-MBR system, the anodic chamber of the MFC is submerged into the MBR under anaerobic conditions and the aeration tank of the MBR was directly used as a cathode chamber. The HF membrane module is fixed in the cathode chamber, where the present electric field plays an important role in this integrated system. It has been reported that the trace electricity can stimulate the microbial growth and resulted in degraded pollutants. Some articles have shown that appropriate electrical currents contributed to the extracellular secretion and biofilms formation [16]. The electrochemically active bacteria (EAB) were enriched in the anode that was beneficial to power output, which could provide more protons to the cathode for the removal of substances, and was helpful for improving the efficiency of wastewater treatment [41]. In addition, the electric field can prevent contaminants from being attached to the membrane, thus mitigating membrane fouling [42].

##### Membrane Bioreactor and Air-Biocathode Microbial Fuel Cell

There are various types of MFC, among which the air cathode MFC is a more promising device to treat wastewater [43], while the MBR system and the air-biocathode MFC is another integrated system which was studied in order to achieve low-cost wastewater treatment (Figure 1B). This system can realize a soluble COD removal rate of 97% [35]. The MFC-MBR system consists of an air-cathode MFC and an anoxic/aerobic membrane reactor in which an additional electric field in MBR is provided by the MFC [44]. The pollutant content of the filter layer in the MFC-MBR integrated system was evidently reduced compared with the C-MBR [45]. The mixing system showed the feasibility of the extraction of energy from wastewater without the additional consumption of any sources of energy [11]. The electric field force contributed to reducing the viscosity of sludge particles, which is also a reason for membrane fouling mitigation [46]. The integrated system uses low-cost materials, making it broad and useful for development prospects in practical applications

##### Flat-Sheet Membrane Bioreactor and Microbial Fuel Cell

Flat-sheet membrane bioreactor has been developed for long times, which improved the processing technology and the stability [47]. It was found to be more effective for the removal of COD, NH_4_^+^-N, and phosphorus (P) [36]. The utilization of MFC integrated with a flat-sheet MBR for wastewater treatment, mitigating membrane fouling, and power generation has been also reported (Figure 1C) [48]. In the integrated system, the flat membrane modules, as cathodes of MFC, maintain the flow of wastewater from the anode chamber to the cathode chamber. The MFC-MBR system for wastewater treatment was a new teachnique.

#### 2.1.2. External Configuration

There is a two-stage combined system for treating wastewater with low energy requirements in the MFC and MBR system [49]. The MFC-MBR coupled system has high flexibility, while MFC/MBR can be operated and managed separately with a low impact on each other. Recently, MBR has been proven to be an advanced approach to achieve high quality treated wastewater as a result of post-processing [50]. Therefore, the effluent from MFC can be further treated by MBR to meet strict discharge standards.

##### Membrane Bioreactor and Sludge Microbial Fuel Cell

The MBR and sludge microbial fuel cell (SMFC) are often studied to treat wastewater. Microorganisms change organic chemical energy directly into useful energy through catalytic reactions in the MFC [51]. During power generation in MFC, the sludge can be hydrolyzed, transformed, and reduced [52]. The sludge extracellular polymeric substances (EPS) accumulate in this system, creating membrane fouling limitation. The SMFC-MBR combined system revealed that the concentration of EPS decreased from 94.2 mg/gSS^−1^ to 75.63 mg/gSS^−1^ after SMFC treatment (Figure 1D) [31]. In this combined system, the removal efficiency of COD and ammonia nitrogen was more than 90%. The sludge reduction rate was also higher than the traditional MBR and the average voltage generated by SMFC was 430mV [37]. Therefore, it was shown that the MFC-MBR combined system has advantages of decomposing sludge and recycling energy [53]. In summary, improvement of wastewater treatment efficiency, low energy consumption, and mitigation of membrane fouling can be obtained by this combined MFC-MBR system [54].

##### Osmotic Membrane Bioreactor and Microbial Fuel Cell

MFC and osmotic membrane bioreactors (OMBR) have been combined to treat wastewater by applying various structures of reactors (Figure 1E) [55]. Osmosis membranes were used in bioreactors to accomplish water rescue in the process of sewage treatment [55]. MFC combined with OMBR was mutually beneficial because the solutes accumulated in the OMBR increased the conductivity and alkalinity of the solution, thereby improving power output from the MFC (3 W/m^3^ goes up to 11.5 W/m^3^). The wastewater was pre-treated to reduce sludge production in MFC and then to reduce membrane fouling in the OMBR [38]. The combined MFC-OMBR system has been investigated in diverse situations such as solute transport, membrane flux level, and nutrient elimination. It has presented the synergistic effect of MFC and OMBR in energy production and sustainable wastewater treatment. This combined system was effective in wastewater treatment, eliminating NH_4_^+^-N, COD, and P, along with with electric energy generation and the reduction of membrane fouling. However, more studies are needed to improve the efficiency of wastewater treatment and alleviate membrane fouling.

##### Anaerobic Fluidized Bed Membrane Bioreactor and Microbial Fuel Cell

Several studies have reported that an anaerobic fluidized bed membrane bioreactor (AFMBR) is a potential wastewater treatment method with a reduction in membrane fouling. Granular activated carbon was used in AFMBR and as a post-treatment system, which helped to reduce membrane fouling [56]. A two-stage coupled system composed of the MFC and AFMBR was developed to treat wastewater (Figure 1F). It is mainly used for the purpose of treating wastewater with a low energy demand and membrane fouling mitigation. This coupled system was reported to operate continuously for 50 days with a constant high osmotic flux, without the requirement of membrane cleaning. The total power of 0.0186 kWh/m^3^ was used in this combined system, which was slightly less than the power generated by MFC [28]. The MFC-AFMBR combined system is not only efficient in treating wastewater, but also has a low energy consumption.

### 2.2. MEC-MBR System Configuration

#### Microbial Electrolysis Cell-Anaerobic Membrane Bioreactor

A MEC and MBR combined system has been used to achieve sustainable wastewater treatment (Figure 1G) [30]. Several studies have shown that the HF membrane in a MEC-Anaerobic membrane bioreactor (AnMBR) system has dual functions in the cathode, namely, H_2_ evolution reaction and the membrane is used to filter and treat wastewater. In this system, with the increase of voltage, the negative charge on the sludge surface increases, which leads to an increase in electrostatic repulsion between sludge particles [46]. Therefore, sludge particles may not be easy to deposit on the membrane and the formation of the sludge cake layer may be inhibited, resulting in a reduced membrane pollution rate [57]. The voltage of 0.6V was applied to MEC-AnMBR for reducing membrane pollution and enhance wastewater treatment efficiency [46]. The application of MEC to MBR properly mitigates membrane fouling. At the appropriate applied voltage, the interaction between electrodes and microbes accelerates the electron transfer rate, potentially increasing the degradation ability of microorganisms, while excessive voltage may damage the cell membrane of microorganisms and inhibit the growth and metabolic rate [57]. Similarly, this system might be an ideal treatment for wastewater.

## 3. Microbial Electrocatalysis Systems Coupled with MBR Enhance the Efficiency of Wastewater Treatment

Microbial electrocatalysis systems for wastewater treatment have been studied over the previous decades. These systems have no widely practical application due to low wastewater treatment efficiency and high costs [26]. There was no obvious development on electrode materials, membrane components, and microbiomes [4]. In recent years, researchers have found that microbial electrocatalysis systems coupled with MBR achieve a “mutual reciprocity and mutual benefit” effect and provides a new method for wastewater treatment.

### 3.1. MFC-MBR Combined System for Wastewater Treatment

The performance of wastewater treatment systems was evaluated by COD, NH_4_^+^-N, and P removal efficiencies [31]. The use of MFC alone results in low processing efficiency and a low quality of the treated wastewater [11]. Non-Pt MFC was used to treat wastewater where the COD and NH_4_^+^-N removal was found to be 77.1% and 80.7%, respectively, with an organic loading rate of 4.9 kg COD/m^3^d [58]. An experimental MBR setup was also constructed to evaluate oil field wastewater treatment efficiency. The organic carbon and COD removal efficiencies were observed to be 92% and 90.9%, respectively [32]. Thus, the MFC-MBR combined system was studied for highly efficient wastewater treatment. The HF membrane was used in this combined system to treat wastewater. It was reported that COD, NH_4_^+^-N, and total nitrogen (TN) removal efficiencies increased by 4.4%, 1.2%, and 10.3%, respectively, in the combined system compared to the C-MBR [31]. Several reports show that a biofilm was formed on stainless steel along with the filtration material and cathode. The COD and NH_4_^+^-N removal efficiency was 92.4% and 95.6%, respectively [59]. Two groups of combined MBR-MFC systems were developed under open-circuit and closed-circuit conditions. The COD removal efficiency was 86.1% and the NH_4_^+^-N removal rate was 97.5% under a closed-circuit [60]. The microbial community was analyzed in biofilm, which showed that the relative abundance of *Lactococcus, Bacillus, Pseudomonas,* and *Saprospiraceae* (uncultured) was 28.3%, 12.3%, 8.8%, and 8.4%, respectively, while in the C-MBR system, the relative abundance of *Pseudomonas, Rhodocyclaceae* (unclassified), *Lactococcus,* and *Comamonas* was 12.5%, 11.9%, 10.1%, and 9.8%, respectively. It was clear that the bacterial community composition was different between the MFC-MBR and C-MBR systems [61]. *Lactococcus* is an electrochemically active gram-positive bacterium that produces various membrane-related Quinone electron receptors that mediate electron transport. The abundance of *Saprospiraceae* (uncultured) and *Bacillus* in MFC-MBR were higher than those in C-MBR. *Saprospiraceae* is related to protein degradation and helps to remove ammonia nitrogen and *Bacillus* appeared as the aerobic nitrification/denitrification genera [61]. In addition, some denitrifying bacteria were abundant in the MFC-MBR system, which might be both stimulated by EAB and beneficial for electron acceptance. In the MFC-MBR system, COD was oxidized at the anode and most of the organic contaminants were eliminated at the cathode compartment [62]. In an internal configuration, COD consumption associated with electrical energy production was presented by the electricity-generating microbes and common microbes, which was stimulated by electricity [37]. In addition, this system has also been studied to treat cheese wastewater and achieved an efficient removal of COD and TN [50]. Anaerobic bacteria in the inner layer of cathode biofilms can use the organic matter in wastewater as denitrifying electron donors, resulting in TN removal [31]. The improvement of NH_4_^+^-N removal efficiency was due to the following reasons: (1) Denitrification can be achieved by nitrate as a terminal electron acceptor at the cathode and EAB, which attaches to the electrodes degrading the organic matter [63]. (2) In the MFC–MBR system, proper current may enrich denitrification, enhancing the activity of denitrifying bacteria [31], which promotes NH_4_^+^-N removal. Therefore, this shows that the higher NH_4_^+^-N removal rate of the MFC-MBR system, achieved through the bio-electrochemical process, affects the denitrifican activity along with the denitrification efficiency [62]. Moreover, wastewater treatment is improved by integrating the MFC-MBR system (Table 2). In an external configuration, MFC and MBR are two separate devices. MFC always plays the role of pre-processing. After the wastewater treatment in MFC, water flows into the MBR for secondary treatment. Compared with MFC or MBR only, the efficiency of wastewater treatment is obviously improved.

### 3.2. MEC-MBR Combined System for Wastewater Treatment

MEC produces clean energy by converting organic matter in wastewater in the form of hydrogen or methane [29,70]. Some papers suggest that MEC cannot be used as an independent technology for urban sewage treatment because it needs a post-treatment or integrated processes to meet discharge limits for the water to be reused. MEC-AnMBR systems have been reported for wastewater treatment [26]. The COD removal efficiency was 52.6% without any applied voltage, but when the voltage was 0.6 V, the COD removal efficiency reached 70.6% [46]. The removal efficiency of COD decreases with the increase of voltage, mainly because higher voltage can lead to more serious plasma rupture, lower microbial growth rates, and lower metabolic activity [71]. In this system, MEC coupled with anaerobic forward osmosis MBR was reported for synthetic wastewater treatment, which gave a COD removal efficiency of 98% [72]. Low currents flowing through biofilms have been shown to have a positive effect on microbial survival and growth [71]. It has been reported that low currents can enhance protein secretion of *Fusarium oxysporum* [73]. Researchers have shown that by using the current densities of 3, 5, and 7 A/m^2^, the relative abundance of some functional bacteria such as *Nitrospiraceae* was 8.5, 12.5, and 12.6%, respectively. At the same time, the relative abundance of *Rhodocyclaceae* was 8.1, 8.8, and 9.7%, respectively. These results show that the removal of nitrogen and phosphorus was 98%, which was higher than the control bioreactor (9.6 and 5.0%, respectively) without current density [74]. When the voltage was 0.7 V, the net energy consumption of the MEC-AnMBR system (0.27 kWh/m^3^) was lower than the energy consumed by aerobic MBR (1–2 kWh/m^3^) [30].

## 4. Microbial Electrocatalysis Systems Coupled with MBR Alleviate Membrane Fouling

Membrane fouling of MBR is attributed to colloids, solutes, cell debris, microorganisms, and biopolymers on the membrane, which lead to membrane pore plugging that declines membrane flux [27]. The membrane fouling mainly includes inorganic fouling, organic fouling, and biofouling. Membrane fouling is one of the reasons limiting the wide application of MBR. It has been reported to mitigate membrane fouling, including surface modification of the membrane [75], the addition of chemicals [76], and aeration [77]. It not only causes environmental pollution but also increases the cost. Researchers have found that soluble microbial products (SMP) and EPS are the most important biological factors that cause membrane fouling [11]. An additional electric field can remove some foulants with negative charges (sludge, SMP, and EPS) from the membrane in the MBR system [78]. In addition, MBR-MFC has potential benefits for achieving energy consumption and recovery, minimizing membrane fouling by improving their performance [79]. The electric energy generated by MFC can alleviate the MBR membrane fouling and partially offset energy consumption. Compared with C-MBR, membrane fouling of the MFC-MBR system was significantly reduced (Table 3). It is a crucial parameter for determining membrane filtration efficiency in the MBR system, which was measured by transmembrane pressure (TMP). Fouling mitigation reported that the internal configuration showed that, after 15 days, the TMP reached 1.2 kpa in the MFC-MBR system, while the TMP reached 1.2 kpa within 4 days in the C-MBR. An electric field forces the foulants with negative charges to go away from the membrane through electrostatic repulsion [62]. In an external configuration, after MFC treatment, there were fewer pollutants in the wastewater flowing into the MBR, so the membrane fouling was alleviated. MFC-MBR system has a certain feasibility to alleviate membrane fouling.

**Figure 2:**
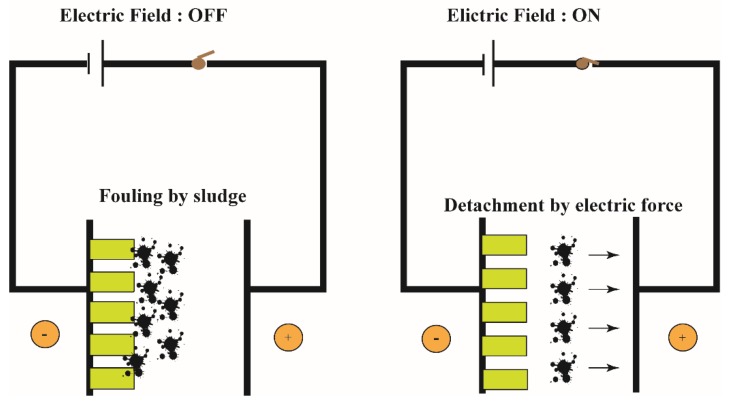
Effect of additional electric field on MBR membrane fouling.

In the MEC-MBR system, the cathode has the function of producing hydrogen and membrane filtration to treat wastewater [80]. The release of hydrogen in the form of bubbles can properly mitigate membrane fouling [30]. In addition, applying voltage to form an electric field force gives the membrane module of the cathode a negative charge that prevents the pollutant with a negative charge from adhering to the membrane module by electrostatic repulsion (Figure 2) [33]. MEC-AnMBR system was reported to remove organic pollutants from wastewater. It was found that the upsurge of applied voltage gradually slows down the membrane fouling rate of the MEC-AnMBR reactor, and the succession of membrane fouling can be prolonged from 60 h to 98 h. The main reason for this is that, with the intensification of applied voltage, the EPS-protein/EPS- polysaccharide ratio was decreased. The increase of applied voltage will cause an increase of the zeta potential absolute value of sludge particles and a decrease in sludge viscosity [46]. The application of electric field forces to mitigate membrane fouling of MBR may be a promising approach.

## 5. Challenges and Future Prospects

Microbial electrocatalysis systems coupled with MBR demonstrate mutual benefits, especially enhanced wastewater treatment efficiency and alleviated membrane fouling. In addition, the voltage generated by MFC can partially offset the energy consumption in the MFC-MBR system. The combined MFC-MBR system is classified into two configurations. Both types illustrate efficient integrated processes for wastewater treatment and have low operating costs. In an internal configuration, the operating parameters and complicated technologies are the primary obstacles for future development. As compared to the external configuration, the internal configuration requires advanced technology, including a more complex design and assembly processes. Although recent studies have shown that the voltage generated by MFC can alleviate membrane fouling in the MBR, membrane fouling is also a major limitation in MBR operation and should be resolved. Some traditional methods used to clean the membrane, like physical washing, showcase a drawback as the fouling rate increases rapidly after cleaning. The membrane needs to keep a certain lifespan, especially for operating an integrated system, this process is time-consuming and expensive and thus is impractical for use in long-term wastewater treatment. It is critical to increase the membrane life by developing novel membrane materials. Biological manipulation and advanced technologies that reduce the cleaning frequency will aid in the commercial application of these systems. In an external configuration, the MFC and MBR work independently. The MFC technology is involved in the first treatment step and the effluent from MFC is treated via a membrane filtration in a conventional MBR. To improve the efficiency of each individual technology, it is crucial for the two technologies to work together. After MFC treatment, the concentration of pollutants before flowing into the MBR for treatment needs to be detected, especially in external systems. Thus, biosensors for water quality monitoring should be developed in MFC-MBR systems. MFC based biosensors can directly provide the electrical signals related to pollutant substrate concentrations. Under the optimal condition the MFC biosensor developed a linear relationship between the voltage output and substrate concentration. The pollutant substrate concentrations can be indicated directly by the voltage. The technology has been studied for water quality monitoring in real-time. The biosensor for COD detection in wastewater has been evaluated, which has the advantages of field implementation, online monitoring, and less chemical addition. It is promising to use biosensors in the MFC-MBR system to detect contaminants. This can reduce the time, energy loss, and maximize the efficiency of both technologies. The MEC-MBR integrated system can achieve mutual benefits in wastewater treatment, including enhanced wastewater treatment and alleviated membrane pollution. However, the application of additional voltage indicates that additional energy input is required. Both MEC and MBR require energy consumption and the absence of aeration at the MEC cathode is not conducive to remove NH_4_^+^-N in the cathode chamber.

The coupled system has not been used for large-scale practical applications. The membrane fouling problem has not been substantially solved by electric field forces. Electric field forces can only alleviate membrane fouling (when the TMP reached 1.2 kpa, CMBR used 4 days while MFC-MBR used 15 days), but cannot solve the membrane fouling problem. Thus, membrane fouling remains an obstacle that needs further study if MFC-MBR/MBR is to be widely used for wastewater treatment. The cost of the MFC/MEC-MBR combined system is large in scale, mainly including electrode (carrier material, catalyst, and current collector), membrane module, and operation/maintenance expenses. Thus, this combined system is still being studied in order to reduce costs and to become more cost-effective. Various studies have focused on improving the performance of the microbial electrocatalysis systems coupled with MBR. With current advances in technologies and materials, the combined system is expected to have a promising future and more attention is required for the improvement of MBR membrane modules. For instance, polyvinylidene fluoride (PVDF) has a better chemical stability and mechanical strength. Nanotechnology is used in PVDF membranes to enhance fouling resistance (PVDF membranes with carboxylated nanodiamonds) [81]. In addition, the selection of electrode materials in microbial electrocatalysis systems is very important, as they can influence biofilm formation, electrical conductivity, corrosion performance, and cost. Synthetic microbiome and biofortification can be used to improve the efficiency of wastewater treatment.

## 6. Conclusions

This review summarized the recent studies about microbial electrocatalysis systems coupled with MBR, describing the strength, stability, and drawbacks, along with the future challenges of the systems. These combined systems not only mitigate membrane fouling, but also have high efficiencies for wastewater treatment. The MFC can generate electricity directly from the wastewater, which can partially offset energy consumption. In addition, functional microbes play an important role throughout the operating system. Although the combined system still has several limitations, the microbial electrocatalysis systems coupled with MBR showed impending advantages as a new method for wastewater treatment.

## Figures and Tables

**Table 1:** The removal efficiencies of COD, NH_4_^+^-N, and P in different microbial electrocatalysis systems coupled with MBR processes.

Reactor	COD Removal Rate	NH_4_^+^-N Removal Rate	P Removal Rate	Reference
Hollow-fiber membrane bioreactor and MFC	>90%	>80%	>65%	[34]
MBR and air-bio cathode microbial fuel cell	97%	97%	-	[35]
Flat-sheet membrane bioreactor and MFC	94.2%	-	75%	[36]
MBR and sludge microbial fuel cell	>90%	>90%	-	[37]
Osmotic membrane bioreactor and MFC	>90%	-	>99%	[38]
Anaerobic fluidized bed MBR and MFC	89 ± 3%	-	-	[39]
MEC and anaerobic membrane bioreactor	96.8%	-	-	[40]

**Table 2:** The COD removal rate and NH_4_^+^-N removal rate in different microbial electrocatalysis systems coupled with MBR processes.

Anode	Cathode	Substrate	Membrane	COD Removal Rate (%)	NH_4_^+^-N Removal Rate (%)	Reference
Graphite rod	Graphite rod	Synthetic wastewater	Fe/PVDF membrane	97.40	96.70	[33]
Graphite rod	Carbon fiber cloth	Synthetic wastewater	MnO2/PVDF membrane	97.00	93.00	[64]
Graphite rod	Carbon fiber cloth	Synthetic wastewater	RGO/PVDF/MnO2 membrane	97.00	-	[65]
Carbon felt	Activated carbon	Synthetic wastewater	PVDF Hollow-fiber membrane	97.00	-	[66]
Graphite rod	Graphite rod	Simulatedwastewater	PVDF/carbon fiber cloth	90.00	80.00	[45]
Carbon fiber cloth	Carbon fiber cloth	Synthetic wastewater	PVDF/carbon fiber cloth	90.00	80.00	[34]
Graphite felt	Stainless steel mesh	Municipal wastewater	Stainless steel mesh Membrane	92.60	96.50	[61]
Graphite rod	Stainless steel mesh	Synthetic Wastewater	Stainless steel mesh Membrane	86.10	97.50	[62]
Graphite granules	Stainless steel mesh	Artificial Wastewater	Stainless steel mesh Membrane	95.30	-	[36]
Graphite granules	Polyester filter cloth,	Municipal wastewater	Polyester filter cloth	95.00	-	[67]
Graphite rod	Stainless steel mesh	Municipal wastewater	Stainless steel mesh Membrane	93.70	96.50	[68]
Carbon brush	Carbon cloth	Domestic wastewater	PVDF Hollow-fiber membrane	90.00	-	[69]
Graphite rod	Stainless steel mesh	Synthetic wastewater	Stainless steel mesh Membrane	92.40	95.60	[59]

**Table 3:** Trans-membrane pressure (TMP) of the MFC-MBR system and C-MBR.

Reactor Type	Anode	Cathode	Membrane	TMP of First Membrane Cleaning (KPa)	Reference
MFC-MBR	Graphitic plate	Graphitic rod	Hollow fiber membrane	21	[11]
C-MBR	Graphitic plate	Graphitic rod	Hollow fiber membrane	40	[11]
MFC-MBR	Iron plates drilled	Flat-sheet conductive membrane module	Flat-sheet conductive membrane module	16	[24]
C-MBR	Iron plates drilled	Flat-sheet conductive membrane module	Flat-sheet conductive membrane module	30	[24]
MFC-MBR	Stainless steel bolt	Carbon brushe	Hollow fiber membrane	6	[31]
C-MBR	Stainless steel bolt	Carbon brushe	Hollow fiber membrane	30	[31]
